# Efficient treatment of a metastatic melanoma patient with a combination of BRAF and MEK inhibitors based on circulating tumor DNA analysis: a case report

**DOI:** 10.1186/s13104-017-2650-5

**Published:** 2017-07-25

**Authors:** Gaelle Quéreux, Guillaume Herbreteau, Anne-Chantal Knol, Audrey Vallée, Amir Khammari, Sandrine Théoleyre, Mélanie Saint-Jean, Brigitte Dréno, Marc G. Denis

**Affiliations:** 1 Department of Dermatology, INSERM CIC1413, Nantes University Hospital, Nantes, France; 2 CRCINA INSERM U1232, Nantes, France; 3 Immuno-Dermatology Laboratory, Nantes University Hospital, Nantes, France; 4 Department of Biochemistry, Nantes University Hospital, 9 quai Moncousu, 44093 Nantes Cedex 01, France

**Keywords:** BRAF, Melanoma, Circulating tumor DNA, Case report

## Abstract

**Background:**

Fixed tissues are the standard samples used in routine practice for molecular testing. But sometimes tissues are lacking or difficult to obtain. In these cases, circulating tumor DNA released from tumor cells can be used as an alternative source of tumor DNA.

**Case presentation:**

We present the case of a 63-year-old Caucasian woman with a metastatic melanoma and a very poor performance status. A plasma sample was tested and the BRAF p.V600E mutation was detected. Based on this result, a treatment combining a BRAF inhibitor and a MEK inhibitor was immediately started. This patient achieved a complete response. In addition, by repeating the plasma test, we could obtain a precise kinetic of release of mutated BRAF DNA in plasma.

**Conclusions:**

We report here for the first time the efficient treatment of a metastatic melanoma patient on the basis of circulating tumor DNA analysis. This urgent treatment provided a dramatic response in a patient with a very poor initial condition. The kinetic data most likely reflect treatment efficacy.

## Background

BRAF inhibitors have revolutionized the treatment of metastatic melanoma in patients presenting a BRAF V600 mutation in their tumor by showing highly significant clinical objective responses [1–5]. These drugs have been approved in many countries for the treatment of patients with unresectable or metastatic melanoma with a BRAF mutation. Therefore, daily practice requires BRAF mutation testing of patients’ tumors.

Fixed tissues (formalin-fixed and paraffin-embedded) are the standard samples used in routine practice for molecular testing. But sometimes tissues are lacking or difficult to obtain due to the metastases’ location, requiring an invasive and potentially harmful procedure. Lastly, test can fail because of low cellularity or insufficient quality of the DNA. In these cases, circulating tumor DNA (ctDNA) released from tumor cells via mechanisms including necrosis and apoptosis [6–8] can be used as an alternative source of tumor DNA for noninvasive identification of biomarkers. In a recent report Tsao et al. concluded that BRAF mutant ctDNA could be used diagnostically where the tumour block was unavailable [9], but this has never been reported.

## Case presentation

**Fig. 1:**
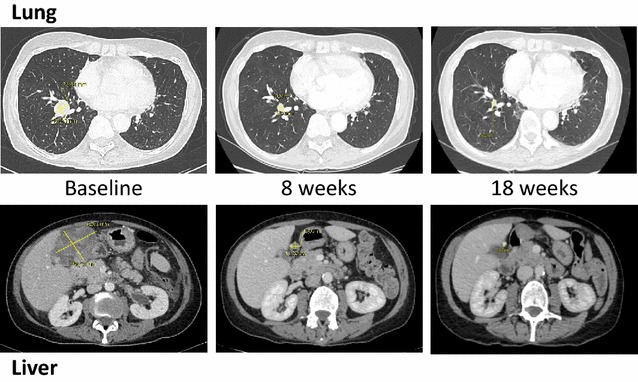
Representative computed tomographic images demonstrating treatment efficacy. Lesions in lung and under liver are presented at baseline and after 8 and 18 weeks of treatment with a combination of BRAF and MEK inhibitors

A 63-year-old Caucasian woman was referred to our unit with a history of rapidly increasing multiple metastases (duodenal, gastric and colon tumors, peritoneal and retroperitoneal carcinomatosis, many lung, bone and gallbladder metastases, mesenteric lymphadenopathies and one brain tumor) (Fig. 1). Biopsies of the colon and stomach concluded the existence of melanoma metastases. The patient had an excision of a primary melanoma of the thigh 15 years earlier but the BRAF status had not been determined. The patient was severely disabled with a very poor performance status (ECOG performance status of 4 and Karnofsky score of 20), ascites and anorexia. Lactate dehydrogenase was equal to 2 times the upper limit.

Because of the seriousness of the metastatic disease and its dramatically rapid progression, supportive care was initially discussed, but we finally decided to test the ctDNA for the presence of a BRAF mutation. Blood was collected, and plasma tested using a ctBRAF mutation test cartridge (Biocartis, Mechelen, Belgium) on an Idylla platform. This very rapid system of ctDNA analysis revealed the presence of the p.V600E mutation in less than 2 h (confirmed 2 weeks later by a test performed on the gastric metastasis using standard techniques [10]). The high concentration of BRAF V600E DNA copies (540 copies/mL plasma) is most likely related to the huge tumor burden [11].

Based on the ctDNA result, a treatment combining a BRAF inhibitor (vemurafenib) and a MEK inhibitor (cobimetinib) was immediately started. After 4 days of treatment, a clinical improvement was noted with a decrease in pain, a progressive recovery of daily living abilities and ascites regression.

**Fig. 2:**
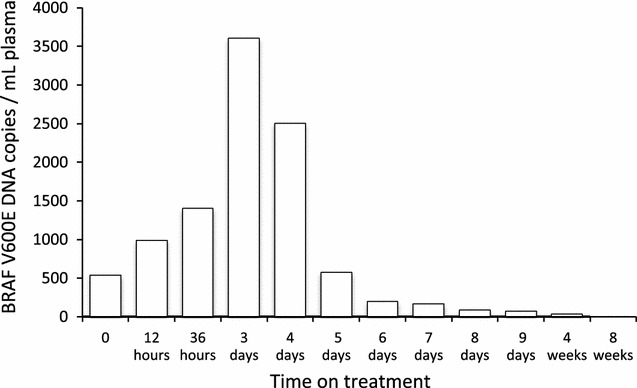
Detection of BRAF V600E mutations in the patient’s plasma. Plasma was collected every day when the patient was in the hospital (9 days), and at each clinical evaluation (after 4 and 8 weeks of treatment). DNA was extracted from plasma (2 mL) using the QIAamp circulating nucleic acid kit (Qiagen). BRAF V600E mutations were detected and quantified by digital PCR using the QuantStudio 3D system and a specific BRAF V600E probe (Thermo Fischer, Courtaboeuf, France)

In parallel, circulating cell-free DNA analysis was repeated to assess the kinetics of its evolution under treatment. DNA was extracted from plasma samples using the QIAamp circulating nucleic acid kit (Qiagen, Courtaboeuf, France), and analyzed by digital PCR using the QuantStudio 3D System and specific probes (Thermo Fischer, Courtaboeuf, France). The level of mutated BRAF DNA increased as early as 12 h after treatment initiation and reached a maximum after 3 days, followed by a significant decrease after day 4 (Fig. 2). These alterations were barely detectable after 4 weeks of treatment and no longer detectable after 8 weeks.

Tumor assessment according to Response Evaluation Criteria in Solid Tumors, version 1.1 [12], performed with a computed tomography scanner, showed a dramatic response after 8 weeks (86% reduction of the tumor burden). With a 20-week follow-up period, treatment was still ongoing and the response maintained [almost complete response observed at week 18: all target and non-target lesions became non-measurable and no new lesions were observed (Fig. 1)].

## Discussion

We report here for the first time the efficient treatment of a metastatic melanoma patient on the basis of ctDNA analysis. We have recently shown that ctDNA samples may be less prone to heterogeneity and provide a better way of determining overall tumor mutation status than a small biopsy [11]. Another potential advantage of ctDNA is that it allows monitoring response [9]. Several reports have shown that, in melanoma patients responding to treatment, a decrease in ctDNA is seen after 1 month [13, 14]. We defined these kinetics more precisely, showing a very rapid increase in mutated ctDNA after treatment initiation, which probably indicates tumor lysis. It was followed by a rapid decrease, and this is most likely associated with response to treatment, as recently demonstrated for non-small cell lung carcinoma (NSCLC) treated with EGFR tyrosine kinase inhibitors [15, 16]. A similar kinetic has been previously reported [9]. Further studies are required to determine whether analysis of a plasma sample as early as 3 or 7 days after treatment initiation could be used to determine whether the patient will respond or not.

Another benefit of a “liquid biopsy” is that turnaround time for ctDNA analysis is expected to be shorter than for tissue genotyping. A prospective analysis of EGFR testing in the ctDNA of patients with newly diagnosed NSCLC recently demonstrated that the median turnaround time for plasma testing was 3 days versus 12 days for tissue genotyping [17].

## Conclusions

In 2016, the European Medicines Agency and the US Food and Drug Administration approved the use of ctDNA for EGFR testing, authorizing treatment of patients with metastatic NSCLC with osimertinib and erlotinib, respectively. Similarly, BRAF ctDNA testing of melanoma patients might also be used as pre-screening before attempting to obtain a result based on tissue samples. If the test is negative, tissue testing should be performed since BRAF mutations are not always detected in plasma of late-stage patients with BRAF mutation-positive tumors [11, 14, 18]. But if the ctDNA analysis is positive, then treatment could be started earlier. In our case, this urgent treatment provided a dramatic response in a patient with a very poor initial condition due to a huge and rapidly growing tumor burden.

## Abbreviations

ctDNA: circulating tumor DNA
EGFR: epidermal growth factor receptor
NSCLC: non-small cell lung cancer
